# It’s Double Edged: The Positive and Negative Relationships Between the Development of Moral Reasoning and Video Game Play Among Adolescents

**DOI:** 10.3389/fpsyg.2019.00028

**Published:** 2019-01-22

**Authors:** Sarah E. Hodge, Jacqui Taylor, John McAlaney

**Affiliations:** Department of Psychology, Bournemouth University, Poole, United Kingdom

**Keywords:** adolescents, moral development, moral reasoning, Kohlberg, video games, computer games, cross-sectional

## Abstract

Due to the concerns over the effects of video game play, this study investigated adolescents’ moral development and their video game play. 166 adolescents aged 11–18 years (*M* = 13.08, *SD* = 1.91) attending an English school completed an online survey, which included a measure of moral development and questions regarding video game play. In contrast to previous research, male participants were found to have significantly (*p* = 0.02) higher moral reasoning scores than females. The results also suggested a transition in moral development, which takes place between the ages of 12–14. The results of moral development and video game played suggested both positive and negative relationships. Regression analysis suggested that there was a significant positive relationship between the more types of game genres played and higher moral scores. Although not significant, the results suggested a trend for the following variables; years playing video games, mature content, engagement, moral narrative, Grand Theft Auto, Call of Duty, and length of time playing video games which all had a negative relationship with moral scores. The implications of these results are discussed with regards to moral education and the variables involved in video game play, including the role of video game content.

## Introduction

Playing video games is a popular pastime, with 26% of under 18 year olds playing video games and the video games industry worth a total of $23.5 billion (statistics from the United States) (Entertainment Software Association [ESA], 2016). Research on video games began, in part, due to violent content increasingly being used and the increasing popularity of video games. As a result concerns regarding the consequences of exposure to violent content, such as associated aggression following playing with violent games (American Psychological Association [APA], 2015). The media in the 1990s started to portray video games as a threat due to vulnerable children and adolescents having access to and playing early video games (McKernan, 2013). The frequent use of excessive violence in video games has become controversial and as such became the focus of research for the next 20 years. However, recent research has started to examine the positive potential influences and relationships that video games may have, such as skill acquisition (Boyle et al., 2016).

Gibbs et al. (1992) developed the Sociomoral Reflection Measure (SRM) to measure moral development based upon Kohlberg’s stage theory (Kohlberg, 1971). This measure of morality categorizes moral reasoning into stages of development. The first two stages transferred well from Kohlberg’s theory into four measurable stages of development (see Table 1). However, changes were made during the development of the SRM, as the last two stages did not transfer well from Kohlberg’s theory and were dropped. Thus the stages range from stage 1 to stage 4 (see Table 1). Gibbs et al. (1992) also changed the name of the levels to mature and immature (known as Moral type A, henceforth Moral A) rather than Kohlberg’s label, conventional level. In addition another type of reasoning was proposed by Gibbs et al. (1992) known as Moral type B (henceforth Moral B). Moral B reflects a different type of moral reasoning. All participants will have a Moral A score (an average stage of development); however, some will also have a Type B. Moral B reasoning suggests an expression of moral principles, as opposed to Moral A which suggests an embedding of the ethical principles from social conventions.

**Table 1:** SRM norms of Moral A adapted from Gibbs et al. (1992).

School age UK (American)	Age	Global stage	Score boundary of global stage	Maturity
Year 5 (fourth grade)	10.05	2	1.75 – 2.25	Immature
Year 7 (sixth grade)	12.06	2(3)	2.26 – 2.49	Immature
Year 9 (eighth grade)	14.11	3(2)	2.50 – 2.74	Immature
Sixth form (high school)	17.30	3	2.75 – 3.25	Mature
University	19.18	3	2.75 – 3.25	Mature
Adult	50.66	4(3)	3.50 – 3.74	Mature


*Adapted from “N, Mean SRM-SF, mean global stage, age, and SES by sample” by Gibbs et al. (1992). *Moral maturity: Measuring the development of sociomoral reflection* p. 40 Copyright 1992 Lawrence Erlbaum Assoicates, Inc.*

Moral B is described as more prescriptive and internal with an awareness of what ought to be (Gibbs et al., 1992). Moral B consists of three components; Balancing, Fundamental Valuing and Conscience. Balancing is shown by individuals recognizing their own as well as others view points for example “treating others how you would like to be treated.” Fundamental Valuing was shown by individuals understanding the intrinsic value of concepts such as promises and life. Conscience was shown by individuals having an awareness of how they would feel about their actions, for example feeling guilty. To have the additional Moral type B, responses had to make reference to at least two of the three moral B components. Moral B components start from transition stage 2/3 to 4. Table 1 shows the average stage of development for the age groups (Gibbs et al., 1992).

Examples of what could be considered amoral behavior can occur when playing video games such as Grand Theft Auto (GTA) (Rockstar, 1997/2015) due to interacting with content such as nudity, prostitution, guns, drug dealing and driving recklessly. Due to this the Entertainment Software Rating Board (Entertainment Software Rating Board [ESRB], 2015) and Pan European Game Information (Pan European Game Information [PEGI], 2015) were created to oversee and label content to support players in their decisions to buy and play games (Kent, 2001). These are also useful resources for understanding content in video games due to the breadth of detail available. Thomas (2006) argues that it is important to consider the role of morality in video games; the act of doing and having the control to do something in a virtual world and the consequences of those actions are different to merely observing them when watching a film. Virtual environments can simulate real or fictional worlds; these worlds can offer many levels of social interaction and Artificial Intelligence with increasing complexity. Additionally many games contain moral narratives, that presents the player with moral choices such as BioShock 1 and 2 (Irrational Games, 2007/2013), where the player decides to “Harvest” (Kill) or “Rescue” (Save) genetically altered female children.

Different measures have been used to define an individual’s video game habits and include experience and exposure to video games, this includes length of time playing video games (Gentile et al., 2011). Many studies have also included favorite games (Bajovic, 2012). However, previous research has tended to focus on a limited number of game play variables. The present study aimed to address this issue by collecting multiple measures. Engagement is a particularly important element of video game play and consists of many components including: immersion, presence, flow, psychological absorption and dissociation (Brockmyer et al., 2009). Engagement is used as a general term to indicate the level of game involvement; however, these components have been criticized for using different definitions. Brockmyer et al. (2009) developed the Game Engagement Questionnaire (GEQ) to combine these components in a measure. Engagement is important to measure as it is a core experience for an individual when playing video games; thus including this variable would be helpful in understanding the video game experience. Moreover engagement may connect to morality and has not been previously researched.

Most research on the psychological effects of video games has investigated violent content therefore much of the research on morality has been limited to focusing on violent video games. Hartmann and Vorderer (2010) examined whether moral disengagement could explain enjoyment of violent content. Moral disengagement is the selective disassociation of behavior that violates an individual’s moral codes (Bandura et al., 1996). The results suggested that the more familiar with the game used in the experiment, the less negative affect and guilt was reported but the greater the enjoyment (Hartmann and Vorderer, 2010).

Joeckel et al. (2012) examined moral decisions in video games using the Moral Foundations Questionnaire (MFQ) (Graham et al., 2008). The authors found that increased moral salience in the video game was associated with decreased moral violations made. This was replicated in a similar study by Joeckel et al. (2013), with the additional finding that enjoyment did not influence moral salience. Similarly research by Weaver and Lewis (2012) found that decisions made when playing Fallout 3 (Bethesda-Softworks, 2008) a Role Playing Game (RPG) with a moral narrative, were similar to real life decisions made on the MFQ. Furthermore Boyan et al. (2015) examined the relationships between the MFQ and the decisions made in video games from the Mass Effect series (Bio-ware, 2007/2012). Participants were gathered from an online forum focused on discussing Mass Effect. The results suggested that only Fairness/Reciprocity, Purity/Sanctity and Harm/Care foundations were correlated with the decisions made in the video games and only care predicted moral decisions. In addition Triberti et al. (2015) found participants’ had a preference for moral positioning in video games; some would prefer to play as evil characters and some as good characters.

Grizzard et al. (2014) using a 2 × 2 design, examined whether behaving immorally in a video game was related to feelings of guilt and moral salience. Participants were either assigned to a memory recall task (either guilt memory or ordinary memory) or a video game which included either a non-guilt inducing level (playing as a terrorist soldier) or a non-guilt inducing level (playing as a United Nations soldier). Following participation in the assigned condition, the MFQ and measure of guilt were also completed. The results suggested participants playing as terrorists felt significantly more guilt than those who played as UN soldiers. This correlated significantly with the MFQ foundations of Harm/Care and Fairness/Reciprocity, but not with loyalty, or authority. The authors argued that this was to be expected, however, given that authority was a theme, as the participants played as soldiers it would have been interesting to have a non-soldier condition to understand the role of authority. The authors suggest that antisocial behavior in video games could relate to prosocial outcomes as the participants who violate the module could become more morally sensitive due to levels of guilt. However, if the module is being activated and stimulated this does not necessarily lead to a change in behavior. For example, whether increased guilt would lead players to stop killing innocent characters in the game cannot be assessed here, as this behavior was not measured. There was also a female sex bias in the sample (71% female); this could have been reflected in the results especially the sex difference in game play (American Psychological Association [APA], 2015; Ferguson et al., 2015). Plus participants’ previous video game play and experience was unclear and this has been suggested to influence results (Hartmann and Vorderer, 2010; Gollwitzer and Melzer, 2012).

Bajovic (2012) examined if playing violent video games is related to moral reasoning and attitude toward violence with eighth grade students (United Kingdom year 9 aged 13–14). Bajovic (2012) used the Sociomoral Reflection Measure-Short Form (SRM-SF) to measure morality. Much of the previous research has examined short-term post-game effects, i.e., moral decisions made in the game (Grizzard et al., 2014), whereas the SRM-SF can measure the development of moral reasoning. Participants were categorized into the violent group by meeting the following criteria: playing 1–3 h every day, one violent game included as a favorite, and the declaration that they played and enjoy violent games. The only variable to correlate negatively with moral scores was the length of time playing violent video games. There were no significant differences between the violent and non-violent group on moral scores. A sex difference was noted in that females spent less time playing video games and played less violent games than males (Bajovic, 2012).

Much of the literature has focused on violent content and in-game decisions; but it is important to consider other content in video games, such as mature content, to understand the potential relationship between morality and exposure to a variety of video game content. A recent model of media consumption and morality suggests that the long-term components of how media is received and appraised, relates to individuals’ selection of media, in this case their video game play (Tamborini, 2011, 2012). Obtaining many video game play variables would also allow differences in game play experiences to be examined e.g., violent and non-violent games, as well as to control for moral/immoral content and differences of experience (and to some extent expertise). As noted by the American Psychological Association there is a need for research focussed specifically on adolescents (American Psychological Association [APA], 2015), as this group make up around a third of gamers (Entertainment Software Association [ESA], 2015, 2016). Consequently the predictive relationship of moral development and video game play is unclear; this study aims to address these gaps by exploring the influences of both playing violent and non-violent video games and as well as self-reported video game play on moral reasoning in adolescents (Hodge, 2018).

## Materials and Methods

### Participants

Ethical approval was obtained from Bournemouth University, Science, Technology and Health Research Ethics Panel, and the study was carried out within accordance with the recommendations of Bournemouth University’s Research Ethics Code of Practice. All participants gave written informed consent in accordance with the Declaration of Helsinki, with written informed consent obtained from parents/guardians for all participants under the age of 16. A total of 166 participants took part in the study, consisting of secondary and sixth form students from United Kingdom school years 7 to 13 (age range 11–18, *M* = 13.08, *SD* = 1.91). There were similar number of males and females (Male 47% Female 53%), 36.1% of the sample entitled to free school^1^ meals. Free school meals (FSM) was taken as measure of Social Economical Status (SES). The majority of the sample had a White Scottish, Irish English or other background 94.0%. One local secondary school was used in the study which included a sixth form.

### Procedure

An online survey tool (Surveymonkey) was used to create an online survey for administration to participants. The survey was piloted to three secondary school pupils before the main administration. The survey took around 40 min to complete and was administered during lessons. The researcher delivered a 10 min presentation to brief students about the research and how to take part in the survey, followed by general information about how students should complete the survey individually. The instructions for the SRM were read aloud with a fictional example used to aid understanding. Finally the first question of the SRM was read aloud for the participants to think about to illustrate that this is the part that required decision making. If the participants were happy they wrote their full name at the start of the survey to consent. The researcher walked around the classroom while the students completed the survey to make sure students taking part could access the link and to offer help where needed. Gibbs et al. (1992) state that when the measure is administered it is helpful to prompt participants to think about why they think the question is important or not, to support scorable answers. The survey was composed of the following three questionnaires.

### Measures

#### Sociomoral Reflection Measure–Short Form (SRM-SF)

This measure was chosen for the present study as it is applicable for use with, a wide age range. Additionally the SRM is not time consuming for administration (completed in about 25 minutes for participants aged 12 years and older). This is less time consuming compared to other similar measures of morality that require moral decisions and evaluation to be made, such as the Moral Judgment interview, which can take over an hour (Colby and Kohlberg, 1987; Gibbs et al., 1992). It also allowed for an individual’s in-depth moral reasoning without the restrictive responses of a tick box. The measure has been used previously in a similar study (Bajovic, 2012, 2013). The measure required participants to type answers for 11 questions covering five moral themes (Gibbs et al., 1992). SRM has good concurrent validity, *r* = 0.69 and test retest reliability *r* = 0.88 (Gibbs et al., 1992).

#### Video Game Play

Video game play was developed and adapted from previous research, into a questionnaire to include a greater range of response options for game play, than has been used in previous research including number of favorite games (Bajovic, 2013). Questions included: favorite games (up to five), number of years playing video games, length of time per week playing video games and number of genres played. The following content variables were extracted from the favorite games listed: Playing Grand Theft Auto (GTA) (Rockstar, 1997/2015) and Call of Duty (COD) (Activision, 2005/2015), Violent, Mature, Moral narrative and Content Rating (mean ESRB and PEGI rating of favorite games; see Table A1, Appendix A).

#### Game Engagement Questionnaire (GEQ)

This measure consisted of 19 questions regarding how the participant usually feels when playing a video game and a score is given to represent the level of engagement (Yes = 2 Maybe = 1 and No = 0). The maximum score on the measure is 38 α = 0.85 (Brockmyer et al., 2009).

### Data

Participants’ responses for each question were categorized into a stage of development and moral type, A or B. The eleven questions are split by themes: questions 1 to 4 Contract and Truth; questions 5 and 6 Affiliation (related to helping family and friends); questions, 7 and 8 Life questions, 9 and 10 Property and Law and finally question 11, Legal Justice. There are four stages of development (1–4) with three transitional stages in between each stage. A response is scored by matching the response to the appropriate Criterion Justification (CJ). The CJ are responses grouped by moral concepts, such as; empathic role taking, intrapersonal approval and prosocial intentions and include sample responses listed below to assist matching; for example “you may become friends” (Gibbs et al., 1992, p. 71). The authors argue that the language used to represent moral reasoning changes with development. For example reasoning starts with absolute notions like “this will happen” and later change to a more relative notion like “this could happen.” Transition stages represented participants starting to develop into the next stage but not fully and still have lower reasoning; for example understanding other behavior (empathic role-taking) but still pragmatic regarding the consequences (advantages). More mature reasoning will start to understand societal implications of actions. Moral B components exist within some of the Moral A CJs. Once the response had been matched to a CJ the highest stage was used and a score was derived by calculating the mean of the highest stage from the eleven questions. This gave an average score of development ranging from 1 to 4. This score could then be matched to a stage (known as a global stage). It should be noted that not all responses could yield a score and were unscorable, such as if the responses were not moral or contained tautologies^2^.

## Results

This study aims to examine the relationships between moral development, video game play and moral scores (SRM) (Hodge et al., 2015).

### Moral Development

Table 2 shows the SRM stages of the sample. The majority of the sample (67.8%) had immature morality and were in stage 2. Only 31.6% participants had mature morality (stage 3 and above).

**Table 2:** The SRM development of the adolescent sample.

Global stage	Score boundary of global stage	Maturity	Frequency (*N* = 133)	Percent %
1	1.00 – 1.25	Immature	0	0
1(2) upper 1	1.26 – 1.49	Immature	0	0
2(1) lower 2	1.50 – 1.74	Immature	1	0.8
2	1.75 – 2.25	Immature	32	24.1
2(3) upper 2	2.26 – 2.49	Immature	29	21.8
3(2) lower 3	2.50 – 2.74	Immature	28	21.1
3	2.75 – 3.25	Mature	39	29.3
3(4) upper 3	3.26 – 2.49	Mature	3	2.3
4(3) lower 4	3.50 – 3.74	Mature	1	0.8
4	3.75 – 4.00	Mature	0	0


*Adapted from “Using the SRM-SF” by Gibbs et al. (1992). Moral maturity: Measuring the development of sociomoral reflection p. 43–57 Copyright 1992 Lawrence Erlbaum Assoicates, Inc.*

**FIGURE 1:**
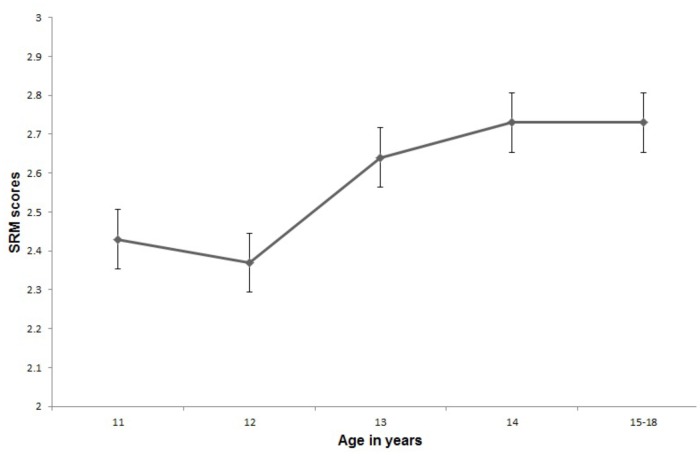
SRM scores of participant by chronological age. A line graph plotting the SRM scores of moral development and age of adolescents, 11–18 years old. Adolescents aged 15–18 were grouped together due to low numbers in the sample.

Figure 1 shows the SRM scores for each of the age groups and suggests that overall moral development is gradual and in the immature stage. Only the 17 year olds had mature morality into stage three. However, 18 year olds were slightly lower and classed as immature but this is likely to be an artifact of the small sample size. There does seem to change between the ages of 12 and 13 years (see Figure 1). A one-way ANOVA^3^ supported this *F*(4,132) = 7.06, *p* < 0.001, and ω^2^ = 0.16, small effect. Gabriels^4^ *post hoc* tests in particular show a change between 12 and 14 years (*p* = 0.002).

### Video Game Play

Table 3 shows there is a sex difference for the continuous video game play variables. Note the large SD for length of time and engagement suggests a lot of variance in these variables. Independent *t*-tests showed a significant sex difference for years playing, number of genres played, Content Rating and Length of time (*p* < 0.01) with medium to large effect sizes but not significant for engagement (*p* > 0.05). Table 4 shows a sex difference for the categorical game play variables. Chi-Squared analysis suggest a significant sex difference for Violent content, Mature content, GTA, COD, Moral narrative (*p* > 0.001), and gaming status (*p* > 0.01). Males were between 7 to 16 times more likely to have to these variables in their game play.

**Table 3:** Descriptive statistics for sex and continuous video game play variables.

Gaming variables continuous		*N*	*M*	*SD*	*t*	df	*r*
Years playing^∗∗∗^ Range = 0–17	Male	56	8.12	3.35			
	Female	52	4.75	2.94			
	Total	108	6.50	3.57	5.53	106	0.47
Genre^∗∗∗^ Range = 0–19	Male	58	8.64	5.37			
	Female	55	5.47	4.14			
	Total	113	7.10	4.94	3.60	108.08	0.33
Content rating^∗∗∗^ Range = 0–5	Male	58	2.95	0.67			
	Female	47	2.09	0.92			
	Total	105	2.57	0.90	5.38	81.51	0.51
Length of time^∗∗∗^ Range = 0–37.5	Male	56	19.37	11.51			
	Female	58	9.19	11.05			
	Total	114	14.19	12.34	4.82	112	0.41
Engagement Range = 0–38	Male	38	20.18	7.51			
	Female	34	16.65	12.42			
	Total	72	18.51	10.21	1.44	53.05	0.19


*r is the effect size reported. *^∗^p* < 0.05, ^∗∗^*p* < 0.01, and ^∗∗∗^*p* < 0.001.*

**Table 4:** Descriptive statistics for sex and categorical video game play variables.

Gaming variables categorical		Yes	No	Total	χ^2^ (1)	Odds ratio
Gaming status^∗∗^	Male	63	0	63		
	Female	61	9	70		
	Total	*124*	*9*	*133*	8.69	9.29
Violent^∗∗∗^	Male	53	5	*58*		
	Female	18	27	*45*		
	Total	71	32	*103*	31.24	15.82
Mature^∗∗∗^	Male	52	6	*58*		
	Female	18	27	*45*		
	Total	71	33	*103*	28.70	12.94
GTA^∗∗∗^	Male	26	32	*58*		
	Female	5	39	*44*		
	Total	31	71	*102*	13.24	6.23
COD^∗∗∗^	Male	36	22	*58*		
	Female	8	36	*44*		
	Total	44	58	*102*	19.65	7.45
Moral Narrative^∗∗∗^	Male	45	13	*58*		
	Female	15	30	*45*		
	Total	60	43	*103*	20.41	6.92


*χ^*2*^: Chi-Squared. Odds ratio is the effect size reported. *^∗^p* < 0.05, ^∗∗^*p* < 0.01, and ^∗∗∗^*p* < 0.001.*

### Moral Development and Video Game Play

Table 5 also suggests that males had higher moral scores than females: males reaching a higher developmental Global stage. This difference was significant *t*(131) = 2.34, *p* = 0.02, *r* = 0.2. The findings for gaming status suggested that participants who played games were a Global stage higher than those who do not play video games. However, the non-gaming group (*n* = 9) was small in comparison to the gaming group (*n* = 124).

**Table 5:** SRM scores, sex and gaming status.

		*N*	*M*	*SD*	Global stage
Sex^∗^	Males	63	2.62	0.38	3(2)
	Females	70	2.47	0.35	2(3)
Gaming status	Yes	124	2.55	0.38	3(2)
	No	9	2.49	0.27	2(3)


*The parentheses for global stage indicates if the score is in the upper or lower score boundary, see Table 2. ^∗^*p* < 0.05.*

Table 6 reports the results of the regression to investigate which found that moral type, sex and genre significantly predicted moral scores. Moral type B significantly predicted higher SRM∖scores than type A. Males significantly predicted higher SRM scores than females. Playing more genres of video games significantly predicted higher SRM scores. Although not significant playing violent game had a positive correlation with higher moral scores whereas mature content, years playing video games, engagement, moral narrative, Grand Theft Auto, Call of Duty, and length of time playing video games had a negative relationship and therefore, lower moral scores (See Table B1, Appendix B).

**Table 6:** Predictors of SRM scores.

Variable^a^	*B*	*SE B*	β
Constant	1.34	0.56	
Moral Type	0.27	0.13	0.27^∗^
Sex	-0.27	0.13	-0.37^∗^
Age	0.04	0.03	0.21
Years playing	-0.03	0.02	-0.27
Genre	0.04	0.01	0.51^∗∗^
Content rating	0.06	0.08	0.15
Violent	-0.58	0.45	-0.72
Mature	0.64	0.45	0.81
Engagement	-0.04	0.05	-0.11
GTA	0.08	0.11	0.10
Moral narrative	0.05	0.15	0.07
COD	0.24	0.13	0.32
Length of time	-0.01	-0.01	-0.16
*R^2^*	0.42^∗∗^		
Δ*R^2^*	0.25^∗∗^		


*Forced entry method was used as no hierarchy was applied to the input of the gaming variables. Preliminary analysis suggested no significant difference for SRM scores with ethnicity and SES and was not included in further analysis. ^*a*^Gaming status was removed by SPSS from the model due to missing cases. Data labels: Moral Type 1 = A; 2 = B. Gender 1 = Male; 2 = Female, Gaming Status, Violent, Mature, Moral Narrative 1 = Yes; 2 = No. ^∗^*p* < 0.05 and ^∗∗^*p* < 0.01.*

## Discussion

This study examined moral development (SRM scores) and video game play. A significant change in moral development was evident in the sample between the ages of 12 and 14. Additionally, it was found that secondary and sixth form students’ moral development is immature and still developing. Interestingly males were found to have higher moral scores than females, in contrast to much previous research which has found that females within this age group have higher levels of moral reasoning (Gibbs et al., 1992). Males were found to play video games for longer than females, and also be more likely to play higher rated and more violent video games. In addition a group of adolescents seemed to be playing video games for an excessive length of time. Although the non-gaming group was small the majority of adolescents did play video games, with the following variables; moral type, sex, and video game genre, found to be significant predictors of moral scores in the regression model.

### Implications

As expected moral type was shown to predict moral scores; moral B predicted higher moral scores. The sex difference in video game play that was found could be connected to the sex difference in morality or alternatively other factors could be of influence. The sex differences were similar to those found by Bajovic (2012) in that females played video games in general less and violent games specifically less often than males, which is consistent with previous research (Gentile et al., 2011; Hartmann et al., 2015). Ferguson et al. (2015) found sex differences with adolescent females, showing they experience more stress from video game play than males. In addition to sex difference this demonstrates the importance of gathering more data about video game play and representing both sexes in research. Individuals who play video games should be categorized by how, what and when they play games. For example it could be the difference between comparing casual game use like Candy Crush and a PC or console title like GTA (Rockstar, 1997/2015); Ferguson (2014) also highlights the importance of this. The prevalence of video game play was further represented by the small number of participants that reported not playing video games (*n* = 9), showing that a high majority of the sample were playing video games, further demonstrating the importance of gathering these data. Conversely, the engagement variable was not significantly different for males and females; this could suggest that the sex difference in video game play could be closing as both were similarly engaged with the game played. Additionally, it could suggest this experience does not differ between the sexes.

The number of genres of video games played was shown to be a significant predictor of higher SRM moral scores. This suggests that certain aspects of game play could have a positive relationship with moral development such as playing a variety of genres of video games. Furthermore, some gaming variables had negative relationships but none were significant predictors of lower moral scores, including; years playing video games, mature content, engagement, moral narrative, GTA, COD, and length of time playing video games. These non-significant variables could suggest that video game play and content may not have a direct relationship with morality. Nevertheless the finding that males had higher video game play consumption and displayed higher moral scores, suggests that video game play could potentially be supporting of moral development, Khoo (2012) argues that playing video games has the potential for individuals to learn skills such as working in teams and could be a tool to assist in moral education. Khoo (2012) applies Kohlberg (1971) moral development theory to video games as some games include guilds which require cooperation. The results of this study connect to this as it could be that guilds and community could stimulate higher moral reasoning, transition stage 3 to stage 4 when individuals start to consider societal implications for reasoning (Gibbs et al., 1992). Alternatively, video games tend to reward certain behaviors (Heron and Belford, 2014), which connects to immature reasoning as right and wrong is determined by reward and punishment.

Another explanation is that that those with higher moral scores, more mature moral reasoning may also be more proficient at morally disengaging through justification, e.g., it is just a game. This is supported by previous research that found that moral disengagement took place in video game to avoid conflicts with enjoyment of the game and with in-game decision making (Hartmann and Vorderer, 2010; Hartmann, 2012). Furthermore, of all the moral disengagement components, moral justification was found to have a very high prevalence in game play (Hartmann et al., 2014). Overall, if video games could be morally stimulating and this is connected to moral development will open many avenues, for future research. For example, if games with a moral narrative activate morality, not only could this be a potential means to get individuals to think about morality in the short term but also activate morality in the long term. Both short term and long term effects of media consumption has been suggested by the Model of Intuitive Morality and Exemplars (Tamborini, 2011). Firstly this could explain the sex difference in moral scores, as games that include a moral narrative were more popular among the males in the sample. Secondly this has implications for how moral development and education for adolescents could be supported.

### Video Game Content

Further research also is needed to examine the trend of violence having a positive relationship with SRM scores and mature content having a negative relationship with SRM scores. This could suggest different types of content have different influences, and perhaps mature content could be of more concern to moral development than violent content. This is interesting as normally games with mature content also contain violent content. Furthermore, violent content was encountered frequently in this study with 68.9%^5^ of the sample listing at least one violent game among their favorites. Bajovic (2012) reported that 86% of participants play violent video games. Kocurek (2012) proposed that violence is a fundamental part of the video game medium. The opposite trend was found in this study with players of violent games having higher moral scores compared to players who only play non-violent titles Bajovic (2012). This is interesting as violent content has been the focus of the media rhetoric on video games, so could it be the case that individuals are desensitized to the violent content and not to the mature content (Carnagey et al., 2007). Additionally this has implications for the other potential content effects of video games and consequently the rating systems (Entertainment Software Rating Board [ESRB], 2015; Pan European Game Information [PEGI], 2015).

The SRM measure has a sub heading of reasoning that includes “prosocial intentions,” research into violent video games and the relationship with prosocial behavior is of current debate (Prot et al., 2014; Ferguson, 2015). Thus it is of note that violent content had a positive relationship with moral scores and mature content had the negative relationship with moral scores. It suggests the potential different effects from types of content such as violent and mature. However, due to the non-significant findings in this study more research is needed to support this. This is particularly since the adolescents in the study were playing video games with a rating higher than their chronological age; this could be influencing moral scores as well as the issue of adolescents playing these games to begin with.

### Limitations of Design

While a cross-sectional design allowed for the data to be collected within the time frame, the limitations are that participants are compared to each other, rather than their own development. Therefore, cause and effect cannot be determined, but used to identify trends for future research. Also only one school was used for data collection; Brugman et al. (2003) found that norms of development are influenced within the school classes and can become similar. The SRM was developed from the constructivist approach, which suggests that environment relates to moral development, hence it is acknowledged that other environmental factors can both contribute and mediate moral development (Gibbs et al., 1992). Some of the unscorable data could be due to participants making quick intuitive moral decisions and as suggested by Haidt and Joseph (2004) this measure may not be sensitive to these types of moral decisions. The number of participants was lower for years 10 and 11 due to parental consent forms not being returned and due to time restrictions and personal choice, the gaming information contained some missing cases. Ethnicity was not considered as the majority of the sample reported a white British Ethnicity. Also one rater was used to code SRM data, it would have been better to have more than one rater to compare coding of the SRM, confirming inter-rating reliability. The GEQ was created to focus on violent video games and could have been restrictive for a general measure of engagement other measures could be considered in future research. Furthermore, emotional experiences and emotions in video games were not measured in this study, and could interact with moral development. Hence, it is suggested how emotions in video game play relate to moral reasoning could be explored in future research.

### Future Research and Conclusion

For moral development, future research could examine finding that of females in secondary and sixth form displayed lower moral scores. In addition, an exploration of whether a change occurs in moral development between the age of 12 and 14 (years 7 and 9) is needed. The results in general suggest, in support of previous studies, that the relationship between morality and video games is a complex one. Further research in this area is needed to gather in-depth gaming information from participants and to investigate variables such as years playing. In addition, the group of adolescents playing video games for an excessive length of time needs further investigation; to examine the role of high game play on development and whether this can become a pathological level of use. These results have broader implications for video game rating systems, moral development and education but also specific implications for parents and the adolescents’ video game play. In sum the results suggested a mixed relationship between video game play and moral development. With further longitudinal research the relationship between moral development and video game play could be discerned.

## Data Availability Statement

The raw data supporting the conclusions of this manuscript will be made available by the authors, without undue reservation, to any qualified researcher.

## Author Contributions

SH contributed to the conception, design, data collection, and analysis. All authors contributed to the manuscript revision, read and approved the submitted version.

## Conflict of Interest Statement

The authors declare that the research was conducted in the absence of any commercial or financial relationships that could be construed as a potential conflict of interest.

## Footnotes

1 42.1% of all pupils were eligible for FSM, which is higher than the national average of 28.5% Ofsted (2015). School Data Dashboard (Online). Available: http://dashboard.ofsted.gov.uk/ (Accessed August 2015).

2 Thirty-three participants produced unscorable SRM responses.

3 Due to low number in the age groups these groups were merged 15–18 for ANOVA.

4 This test was chosen as the group sizes were uneven.

5 This is the percent from number of participants that responded to the question.

## Appendix A

**Table A1:** Rating scale of video game content from ESRB and PEGI.

Scale	ESRB	PEGI
0	Early childhood	N/A
1	Everyone	3
2	Everyone +10	7
3	Teen	12
4	Mature	16–18
5	Adult only	N/A

## Appendix B

**Table B1:** Correlations matrix of SRM scores, demographics and game play variables.

	1	2	3	4	5	6	7	8	9	10	11	12	13	14
(1) SRM	–													
(2) Moral type	0.29^∗∗∗^	–												
(3) Sex	-0.20^∗^	0.00	–											
(4) Age	0.36^∗∗∗^	0.19^∗^	-0.20^∗^	–										
(5) Years playing	0.26^∗∗^	0.24^∗∗^	-0.47^∗∗∗^	0.51^∗∗∗^	–									
(6) Genre	0.38^∗∗∗^	0.19^∗^	-0.32^∗∗∗^	0.24^∗∗^	0.57^∗∗∗^	–								
(7) Content rating	0.08	-0.05	-0.48^∗∗∗^	0.13	0.15	0.28^∗∗^	–							
(8) Violent	-0.11	0.08	0.55^∗∗∗^	-0.21^∗^	-0.31^∗∗^	-0.47^∗∗∗^	-0.76^∗∗∗^	–						
(9) Mature	-0.06	0.07	0.53^∗∗∗^	-0.20^∗^	-0.28^∗∗^	-0.44^∗∗∗^	-0.76^∗∗∗^	0.98^∗∗∗^	–					
(10) Engagement	-0.05	0.17	-0.17	-0.20^∗^	0.30^∗^	0.34^∗∗^	0.18	-0.10	-0.09	–				
(11) GTA	0.02	0.03	0.36^∗∗∗^	-0.14	-0.16	-0.18^∗^	-0.27^∗∗^	0.44^∗∗∗^	0.45^∗∗∗^	0.10	–			
(12) Moral narrative	-0.06	0.13	0.45^∗∗∗^	-0.22^∗^	-0.19^∗^	-0.34^∗∗∗^	-0.62^∗∗∗^	0.71^∗∗∗^	0.73^∗∗∗^	0.02	0.55^∗∗∗^	–		
(13) COD	0.12	-0.01	0.44^∗∗∗^	0.07	-0.02	-0.22^∗^	-0.54^∗∗∗^	0.58^∗∗∗^	0.59^∗∗∗^	-0.26^∗^	0.24^∗∗^	0.29^∗∗^	–	
(14) Amount of time	0.12	0.20^∗^	-0.41^∗∗∗^	-0.11	0.36^∗∗∗^	0.46^∗∗∗^	0.27^∗∗^	-0.26^∗∗^	-0.24^∗∗^	0.38^∗∗^	-0.11	-0.26^∗∗^	-0.04	–
(15) Gaming status	-0.04	-0.12	0.26^∗∗^	0.10	-0.36^∗∗∗^	-0.14	-0.28^∗∗^	0.00	0.00	-0.38^∗∗∗^	0.00	0.00	0.00	-0.30^∗∗^

*^∗^p < 0.05, ^∗∗^*p* < 0.01, and ^∗∗∗^*p* < 0.001.*
